# Effects of depression and anxiety symptoms on cognitive inhibition: A cross-sectional study of structural and functional MRI evidence

**DOI:** 10.1097/MD.0000000000042000

**Published:** 2025-04-11

**Authors:** Maede Bahri, Hassan Farrahi, Maryam Bahri, Hami Mahdavinataj, Seyed Amir Hossein Batouli

**Affiliations:** a Department of Neuroscience and Addiction Studies, School of Advanced Technologies in Medicine, Tehran University of Medical Sciences, Tehran, Iran; b Department of Psychiatry, Kavosh Cognitive Behavior Sciences and Addiction Research Center, School of Medicine, Guilan University of Medical Sciences, Rasht, Iran; c BrainEE Research Group, Tehran University of Medical Sciences, Tehran, Iran.

**Keywords:** anxiety, cognitive inhibition, depression, resting‐state network, voxel‐based morphometry

## Abstract

Evidence shows that depression and anxiety symptoms are associated with reduced cognitive inhibition. Nevertheless, the neural substrates responsible for the effects of depression and anxiety symptoms on cognitive inhibition are yet to be determined. This cross-sectional study adhered to the strengthening the reporting of observational studies in epidemiology (STROBE) checklist. Data from 242 participants from the Iranian brain imaging database were used in this study. To address the neural substrates of depression and anxiety responsible for inhibition, voxel-based morphometry (VBM) analysis and resting-state functional magnetic resonance imaging (RS-fMRI) were used. The depression anxiety stress scale was used to evaluate symptoms of depression and anxiety, and the Stroop test was used for cognitive inhibition. The behavioral results demonstrated that inhibition was significantly negatively correlated with depression and anxiety. The VBM results showed that depression was negatively correlated with gray matter (GM) volume in the left pallidum and the right cerebellum cortex. Additionally, anxiety negatively correlated with GM volume in the left and right cerebellum cortex. RS-fMRI results showed that the thalamus network was positively correlated with depression and anxiety. more importantly, mediation analysis revealed that the right cerebellum cortex and thalamic resting-state network through depression and anxiety had a total indirect effect on inhibition. Clarifying the neural substrates responsible for how depression and anxiety symptoms affect cognitive inhibition could have important implications for interventions aimed at supporting individuals’ cognitive health.

## 1. Introduction

Cognitive inhibition refers to the intentional or unintentional mental process of blocking or tuning out stimuli or information that is not relevant to the task at hand.^[1]^ Inhibition is considered a key cognitive skill in our daily lives, because many successful behaviors depend on it. For example, cognitive inhibition allows people to focus on reading a book, driving, etc, instead of focusing on disturbing noises, past memories, street noises, and so on. Previous research has reported that cognitive inhibition as an essential process in emotion regulation is impaired in anxiety and depression.^[2]^ A deficiency in cognitive inhibition leads to the inability to prevent the negative aspects of information from entering the working memory and then leads to the attention and processing of negative content. In this case, the person enters a vicious cycle that can turn into an anxiety or depressive disorder over time.^[3]^ In the present study, we examined potential neural correlates through which depression and anxiety symptoms may modulate cognitive inhibition. Specifically, we identified how depression and anxiety symptoms affect activity in resting-state networks and gray matter (GM) volume in the brain regions. We then further examined the neural substrates responsible for how depression and anxiety symptoms affect cognitive inhibition.

### 1.1. Cognitive inhibition and symptoms of anxiety and depression

Changes in the intensity, duration, and frequency of cognitive inhibition can affect daily life. In other words, a deficiency in cognitive inhibition affects learning, retrieval, working memory, and comprehension of people and can lead to inappropriate production of incorrect answers.^[4]^ Therefore, with deficient cognitive inhibition, an individual’s ability to suppress the processing of irrelevant stimuli is reduced, and the individual’s ability to respond adaptively to stressful factors is impaired.^[5]^

In general, cognitive inhibition involves the dorsal frontal inhibitory system, and its deficit is a characteristic of psychiatric disorders.^[6]^ A growing body of research has reported cognitive inhibition deficits among individuals with symptoms of anxiety and depression.^[5,7]^ In the clinical literature, affective disorders are associated with impaired concentration and unwanted thinking. For example, anxiety is characterized by uncontrollable worry,^[8]^ and depression by impaired concentration and rumination.^[9]^ Given that cognitive inhibition helps suppress unwanted thoughts and disturbing stimuli, its deficiency creates a vicious cycle in anxious and depressed individuals. In this way, with impaired inhibition performance, long processing of negative information creates a stable negative mood in individuals, which in turn prevents changing and improving mood.^[2,10]^ In other words, deficits in inhibition are associated with increased rumination in depressed people^[11]^ and increased uncontrollable worry in anxious people.^[12]^

### 1.2. Neural correlate of depression and anxiety symptoms

Magnetic resonance imaging (MRI) is used to investigate the pathological structural changes in the brain anatomy related to anxiety and depression. Several studies have reported significant changes in the GM of various brain regions in patients with depression and anxiety disorders, including the frontal lobe, hippocampus, parahippocampal gyrus, lentiform nucleus, temporal lobe, amygdala, and thalamus.^[13,14]^ Neuroimaging studies support a change in the brain of people with symptoms of depression and anxiety - at a subclinical level.^[15]^ Of course, the results related to brain structural changes in people with symptoms of depression and anxiety are contradictory due to different demographic characteristics. For example, 2 studies reported reduced hippocampal volume in subjects with subclinical depressive symptoms.^[16,17]^ Another study reported a reduction in the amygdala volume in people with anxiety symptoms.^[18]^ In addition to these regions, studies have reported numerous structural changes including the anterior cingulate cortex,^[19]^ orbitofrontal cortex,^[20]^ middle temporal gyrus, Rolandic operculum,^[21]^ thalamus, and hypothalamus.^[22]^

In addition to structural changes in depression and anxiety, there is also a disturbance in the level of brain networks.^[23]^ For example, the involvement of networks such as default mode network, cognitive control network, salience network (SN) and ventral attention network has been reported in depression and anxiety.^[23,24]^

### 1.3. Related work

Research has consistently shown a link between cognitive inhibition and symptoms of anxiety and depression^[7,12,25–28]^ as well as the neural correlates associated with these symptoms.^[15–24]^ For instance, research by Burdette et al^[25]^ found that depressive and anxiety symptoms were positively associated with failed inhibition. Similarly, Hallion et al^[12]^ reported a positive association between generalized anxiety disorder (GAD) and impairment in cognitive inhibition. On the other hand, neuroimaging studies have reported pathological structural changes in several brain regions associated with symptoms of anxiety and depression.^[15–24]^ In addition, studies report brain regions that overlap in inhibitory control and emotion regulation, including the dorsal cingulate cortex, anterior insula, inferior and middle frontal gyri, and anterior temporoparietal junction.^[29,30]^ A study by Thai et al^[31]^ found that neural markers of inhibitory control are predictive of treatment outcomes in depression, highlighting the role of inhibitory control in symptom improvement. Despite such studies, the neural substrates responsible for the effects of depression and anxiety symptoms on cognitive inhibition have yet to be determined. In general, the search for the effect of depression and anxiety symptoms on cognitive inhibition has brought diverse studies. These include studies that focus on depressive and anxiety disorders rather than symptoms, studies that use different cognitive tasks to examine cognitive inhibition, and studies that use samples with a narrow age range or small samples. To the best of our knowledge, this study is the first to examine the neural substrates responsible for the effects of depression and anxiety symptoms on cognitive inhibition based on testing a mediation model.

### 1.4. System overview

Using structural MRI and resting fMRI techniques, the current study attempted to examine the effect of depression and anxiety symptoms on cognitive inhibition. It is known that the neural underpinnings of mental state parameters such as cognitive and affective parameters can be investigated using techniques such as voxel-based morphometry (VBM) and resting-state functional MRI. In order to achieve the purpose of the research here, MRI data analysis was used with the aim of extracting resting-state networks as well as brain structure using VBM. Overall, in this study, the data of 242 people examined in terms of physical and mental health were used. The depression anxiety stress scale (DASS) was used to evaluate symptoms of depression and anxiety, and the Stroop test was used for cognitive inhibition.

### 1.5. The present study

Given that depressive and anxiety symptoms can be associated with reduced cognitive inhibition, and subsequently, given the importance of cognitive inhibition as an important cognitive function on individuals’ well-being, this study investigated the neural substrates responsible for the impact of depression and anxiety symptoms on cognitive inhibition.

## 2. Methods

### 2.1. Participants and procedure

This was a cross-sectional study that used data from the Iranian brain imaging database (IBID).^[32]^ In total, the data of 300 participants were used in the study. Of the participants, 19% (58) had a lot of missing responses and were excluded from subsequent analysis. After the exclusion of these subjects, the final sample of 242 participants ranging in age from 20 to 70 years was investigated with a mental health test, the DASS, which provides the level of depression and anxiety, and a cognitive task, the Stroop test, which provides the skill of inhibition. In addition, 2 MRI protocols were investigated.

**Figure 1.**
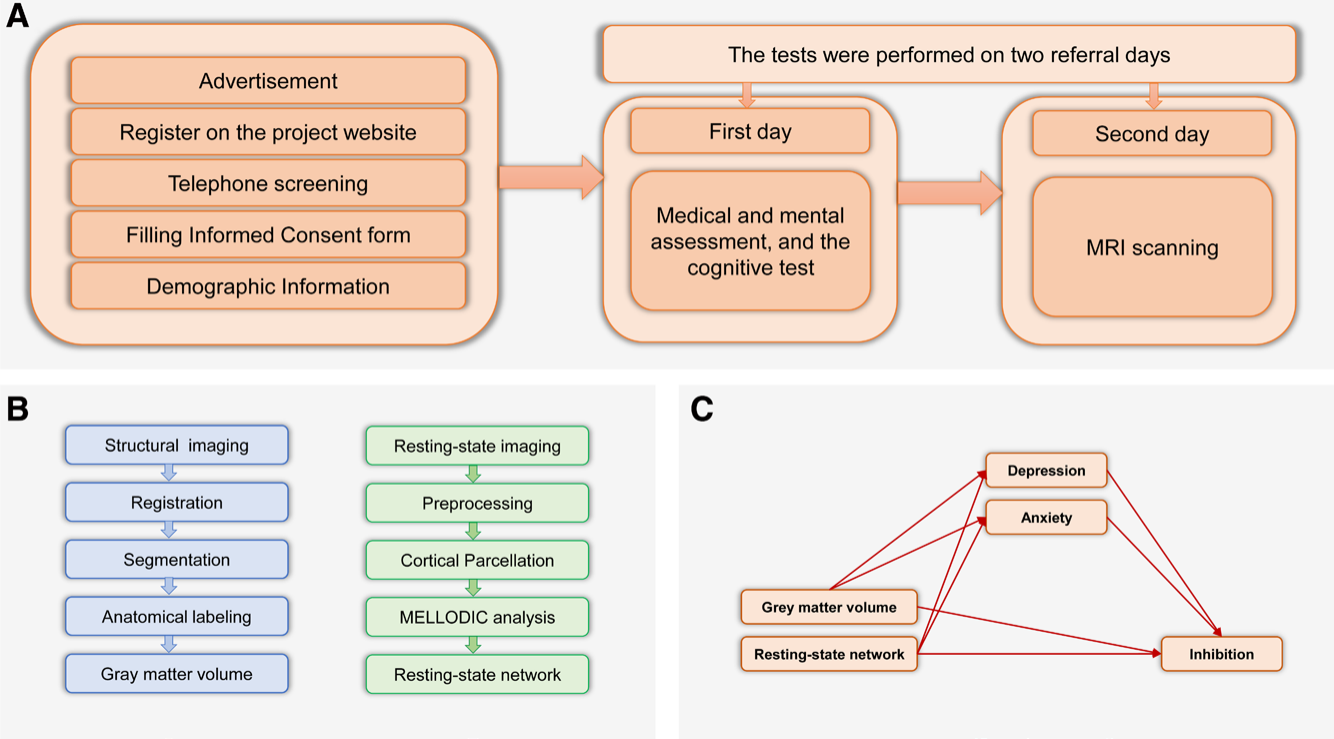
A schematic overview of the experimental design and analysis process. (a) IBID study design for performing experiments. (b) Analysis process for brain imaging measures. (c) Initial hypothetical model. IBID = the Iranian brain imaging database.

The inclusion criteria required in the IBID study included the following: aged 20 to 70 years, with a minimum of 12 years of education, having reading skills, consent to participate in the research, Iranian nationality, and Persian as the first or second mother tongue. Also, the exclusion criteria included neurological disorders or severe somatic problems; such as multiple sclerosis, stroke, epilepsy, migraine, Alzheimer, head trauma, encephalitis, meningitis, cardiovascular diseases, asthma, high blood pressure, high cholesterol, HIV+, liver disease, and hepatitis, weight > 110 kg, pregnancy or breastfeeding, claustrophobia, any history of drug use related to neurological disorders or a prolonged history of drug use (except antibiotics, vitamins, aspirin, anti-nausea drugs, pain relievers, sleeping pills, and vaccinations), and drug use or alcohol addiction (only based on the subjective report). The methods and approaches used to collect the data for the IBID study have previously been described in greater detail^[32]^ (see Figs. 1A and B).

### 2.2. Measures

#### 2.2.1. Mental health tests

The depression anxiety stress scale (DASS-21) was used as a valid and reliable test for measuring the severity of the symptoms of depression, anxiety, or stress.^[33]^ Especially noteworthy is that the DASS is not a clinical instrument and is incapable of diagnosing depression, anxiety, or stress disorder. The short form of this self-report scale has 21 items and 3 main subscales (depression, anxiety, and stress) each with 7 questions. Participants were asked to evaluate each item using a 4-point Likert scale (0 = I strongly disagree, to 3 = I totally agree), indicating the extent to which the statement described their negative emotional experiences in the last week. The Cronbach alpha coefficient was.93 in the Persian version of DASS-21,^[34]^ and.86 in the present study.

#### 2.2.2. Cognitive tests

The Stroop color and word test (SCWT) test was used to measure inhibition.^[35]^ The SCWT as a neuropsychological test, in addition to being widely used to measure cognitive inhibition, is also used to measure several other cognitive functions such as cognitive flexibility, working memory, attention, and processing speed.^[36]^ In this computerized task, the participant is presented with a list of words yellow, red, blue, and green; in half of the trials, words with congruent colors (red, yellow, green, blue) are presented, and in the other half, words with incongruent colors (pink, purple, orange, etc) are presented. Each word is presented in the middle of the screen for 2000 milliseconds, and the distance between each stimulus is 800 milliseconds. The participant is asked to ignore the word and instead press the corresponding color key on the computer system. At the end of the test, the interference score is calculated. The interference score is the result of the difference between the correct congruent answer and the correct incongruent answer.

### 2.3. MRI scanning

The MRI machine utilized in this study was a Siemens 3.0 Tesla scanner (Prisma model, 2016), dedicated to research at the Iranian National Brain Mapping Lab (www.nbml.ir). This advanced scanner features a 50-cm field of view (FOV) with exceptional homogeneity, a whole-body superconductive zero helium oil-off 3T magnet, and a direct connection for head and neck imaging with 20 receive channels. For our research, we employed a 64-channel head coil, which enhances imaging quality and participant comfort. The MRI protocols were chosen to align with established standards from international projects, including the UK Biobank and the ENIGMA consortium. The specific protocols used are detailed below:

Resting-state fMRI data were acquired over 6 minutes using the following parameters: TR = 2500 ms; TE = 30 ms; flip angle = 90°; voxel size = 3.0 × 3.0 × 3.0 mm; 40 slices; matrix size = 64 × 64 × 40; distance factor = 0%; phase encoding direction = anterior to posterior; averages = 1; delay in TR = 0 s; multi-slice mode = interleaved.

T1-weighted MP-RAGE parameters: Acquisition time (TA) = 4:12 minutes; TR = 1800 ms; TE = 3.53 ms; TI = 1100 ms; flip angle = 7°; voxel size = 1.0 × 1.0 × 1.0 mm; multi-slice mode = sequential; FOV read = 256 mm; number of slices = 160; phase encoding direction = anterior to posterior; matrix size = 256 × 256 × 160; averages = 1.

All MRI data were carefully inspected to ensure high-quality imaging. This process involved evaluating key image parameters, including matrix and voxel sizes, verifying the number of time points for resting-state fMRI, and confirming proper right-to-left orientation. Additionally, a detailed visual assessment was conducted to identify potential issues such as macroscopic artifacts, evidence of vibration or motion, improper head positioning or tilt, signal loss, ghosting, and any other artifacts that could affect data integrity. This rigorous quality control step was essential to ensure the reliability of the data for further analysis.

### 2.4. Data analysis

#### 2.4.1. Resting fMRI data analysis

Comprehensive details of our data analysis were published earlier.^[37]^ In summary, the fMRI data underwent a series of 6 preprocessing steps: slice timing correction, realignment, co-registration, normalization, smoothing, and segmentation. The slice timing section was conducted with the following parameters: a total of 43 slices, a repetition time (TR) of 2500 ms, and an acquisition time (TA) of 0.9768 seconds, calculated as (1–1/43). The parameters for realignment were set as follows: quality at 0.9, separation at 4, smoothing at 5, and interpolation at 5. The co-registration settings were as follows: we selected the T1 image as the reference image and included all volumes of the resting-state images as the source images. In the normalization process, we chose the T1 image to ensure proper alignment of the images, and for image to write, We chose all volumes of the resting-state images that were obtained from the final preprocessing step, which involved co-registration. The smoothing parameters were set as follows: FWHM = 6, data type = consistent, and implicit masking = none.

Also, 1 preprocessing step was performed on the structural T1-weighted images, which included removing the skull and non-brain tissues from the T1-weighted brain images. FSL (FMRIB Software Library v6.0 Created by the Analysis Group, FMRIB, Oxford, UK.) offers a tool known as BET (Brain Extraction Tool) for brain extraction tasks. In our analysis, we utilized the BET GUI with the following settings: a fractional intensity threshold of 0.35 and enabled options for bias field correction and neck cleanup.

We employed the MELODIC toolbox (multivariate exploratory linear optimized decomposition into independent components [ICs]), a component of the FMRIB Software Library (FSL v6.0), to analyze brain activation maps during the resting-state. This involved applying spatial independent component analysis (ICA) to decompose 4D datasets into separate spatial and temporal components, which are identified as ICs representing brain activations.

The preprocessed data were imported into MELODIC for group ICA using the temporal concatenation approach, allowing the identification of various activation and artifactual components without requiring a predefined time series model. The MELODIC analysis was configured with the following settings: 63 inputs, interleaved slice timing correction, motion correction using MCFLIRT, spatial smoothing with a full width at half maximum (FWHM) of 5 mm, activation intensity normalization, multi-session temporal concatenation mode, and a threshold for IC maps set to 0.9. The temporal concatenation approach yielded 109 ICs from a total of 242 fMRI datasets.

**Figure 2.**
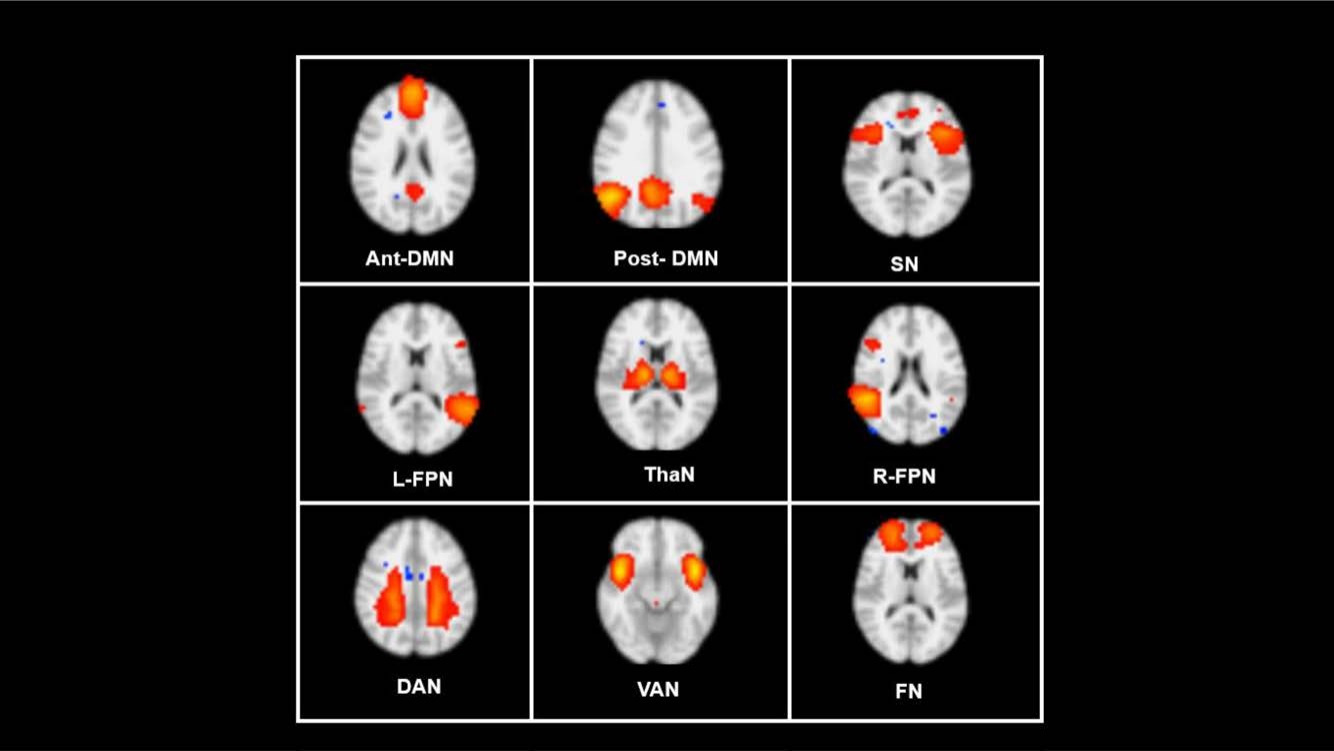
Resting-state networks. Ant-DMN = anterior default mode network, DAN = dorsal attentional network, FN = frontal network, L-FPN = left frontoparietal network, Post-DMN = posterior default mode network, R-FPN = right frontoparietal network, SN = salience network, VAN = ventral attentional network, ThaN = thalamus network.

These 109 components are incorporated into the maps that correspond to real brain activations and reflect the intrinsic activities of individuals during the resting-state, along with the maps pertinent to the artifacts and any other influencing factors. Building on our hypothesis in this study, as well as insights from previous research,^[38]^ We identified the following functional networks from our results, and subsequent analysis steps were conducted solely on these networks: anterior default mode network (Ant-DMN), posterior default mode network (Post-DMN), SN, right and left frontoparietal network (R-FPN & L-FPN), frontal network (FN), ventral attentional network (VAN), dorsal attentional network (DAN), and thalamus network (ThaN). The illustration of this specific resting-state network is presented in Figure. 2.

Dual regression is a valuable tool in group-level resting-state analysis, enabling the identification of subject-specific contributions to group-level ICA. This method generates subject-specific spatial maps and time courses for each group-level component, facilitating comparisons across subjects and groups. We performed dual regression using FSL software, covering all necessary steps. The process was executed via a simple script in a Linux virtual machine running within a Windows environment. Specifically, dual regression was applied to the outputs of MELODIC ICA, which had estimated 114 components for all 242 participants collectively. The resulting outputs were used to quantify the activation strength of each of the 9 networks in the 242 study participants. Activation strength was defined as the average z-value of voxels activated within the network, with activation determined by a z-value exceeding 2.3.

#### 2.4.2. Volumetric data analysis

The specifics of our volumetric analysis methods have been detailed in a previous publication.^[39]^ In summary, the quality of the T1-weighted scans was initially visually assessed to ensure correct orientation and appropriate matrix and voxel sizes. This visual inspection aimed to detect any macroscopic artifacts, signs of vibration, or motion in the images. The assessment ensured an optimal signal-to-noise ratio while evaluating factors such as head tilt, positioning, signal loss, ghosting, and other potential artifacts present in the data.

Subsequently, VBM analysis^[40]^ was conducted as follows. The T1-weighted scans were segmented into GM, white matter (WM), and cerebrospinal fluid (CSF) using the Segment toolbox in SPM12. This process generated native space outputs and utilized data imported from Diffeomorphic Anatomical Registration Through Exponentiated Lie Algebra (DARTEL).^[41]^ The “Run DARTEL” option was employed with default settings, incorporating the “create a template” toolbox to enhance inter-subject alignment accuracy. This involved iteratively averaging DARTEL-imported data for GM and WM tissue types, resulting in the creation of population-specific templates. Following template creation, all GM and WM images were normalized to the Montreal Neurological Institute (MNI) standard space using the “Normalize to MNI space” toolbox.

The objective of this analysis was to estimate the volume of several brain regions. To achieve this, 2 brain atlases were utilized: the Desikan–Killiany Atlas^[42]^ and the Aseg Atlas.^[43]^ The atlases provided the regions of interest for brain areas. The volume of each region of interest (ROI) was determined by summing the probability estimates from both the GM and WM maps. Subsequently, the resulting value was multiplied by the volume of a single voxel, which is 3.375 mm³, using a custom MATLAB script. This process enabled the estimation of the volume for 173 brain regions across all 242 participants.

In the present study, we included 173 GM volumes and 9 resting-state networks.

#### 2.4.3. Statistical analysis

The collected data were analyzed using SPSS (version 26, Chicago) and AMOS (version 24). Initially, frequency, average, and percentage were utilized to elucidate the demographic characteristics of the participants. Next, to conduct the first-level analysis, the mean and standard deviation of each study variable were calculated and reported. To assess the normality of the data, skewness and kurtosis were also evaluated, indicating that the data followed a normal distribution.

Path analysis was employed to elucidate the mediating effects of depression and anxiety. In the second-level analysis, the final experimental model was tested by examining the correlations between variables along the paths of the initial hypothetical model. Nonsignificant paths were systematically removed in a step-by-step process. As a result, the data was subjected to a series of Pearson correlation analyses. Our initial analysis investigated the correlations among depression, anxiety, stress, and inhibition. Next, the study examined the relationship between GM volumes and resting-state networks, along with the role of inhibition. Subsequently, an examination was conducted to assess the correlation between brain structure and function and depression and anxiety. In our model, GM volume and resting-state networks served as independent variables, depression and anxiety acted as mediators, and inhibition was the dependent variable (see Fig. 1C). To control for their potential confounding effects, age and years of education were included as variables in the analysis.

## 3. Results

### 3.1. Preliminary analyses

We used 300 participants of the IBID study in this work. Of these subjects, 58 individuals were removed from the analysis due to numerous missing responses. Overall, around 81% of the participants submitted complete data. The final group for analysis comprised 242 individuals.

The participants were placed in the following 5 age groups: 103 early adults from 20 to 30 years (68 female), 128 early middle-aged adults from 30 to 40 years (89 female), 128 late middle-aged adults from 40 to 50 years (89 female), 128 late adults from 50 to 60 years (89 female), and 106 older adults from 60 to 70 years (68 female). The demographic characteristics of the study participants, stratified by age group, are detailed in Table 1.

**Table 1:** Participant demographic information.

Age group (yr)	n		Age mean (SD)	YoE
	Male	Female		
20 to 30	25	29	25.9 (2.92)	16.79
30.01 to 40	31	33	35.34 (3.15)	16.82
40.01 to 50	24	31	45.67 (2.98)	14.55
50.01 to 60	20	25	54.67 (2.98)	14.11
60.01 to 70	12	12	65.63 (3.51)	14.17

n = The number of participants, SD = standard deviation, YoE = years of education.

#### 3.1.1. Behavioral results

Descriptive statistics for each studied variable (depression, anxiety, and stress and the interference as the index of inhibition) were calculated and presented in Table 2. The kurtosis and skewness (in the range of ± 2) for the studied variables revealed that the data has a normal distribution.^[44]^

**Table 2:** Descriptive statistics and inter-correlations of study variables.

	Mean	SD	Range	Skewness	Kurtosis	Correlation with inhibition
Inhibition	100.67	40.00	17 to 326	−.02	−.21	1
Depression	7.87	8.73	0 to 38	.37	−.45	.15*
Anxiety	5.59	6.96	0 to 42	.36	−.56	.19**
Stress	11.28	9.44	0 to 42	.15	−.48	.07

SD = standard deviation.

* *P* < .05.

** *P* < .01.

We then executed a Pearson correlation analysis which directed that interference as the index of inhibition was correlated with depression and anxiety. It should be noted that there was no correlation between stress and inhibition. The correlation analysis results are detailed in Table 2.

Lastly, age, gender, and years of education were considered as intervening variables. No gender differences were found in the inhibition (*t* (df = 240) = .87, *P* = .38), or in the scores of the depression (*t* (df = 240) = −1.56, *P* = .12), anxiety (*t* (df = 240) = -.61, *P* = .54), and stress (*t* (df = 240) = -.16, *P* = .87). Also, Pearson correlation analysis showed that age significantly correlated with inhibition (*r* = .42, *P* < .001). There was no significant correlation found between age and the levels of depression, anxiety, and stress. Notably, a significant negative correlation was observed between years of education and the study variables. Detailed results are summarized in Table 3. The analyses suggested that age and years of education can act as intervening variables in the context of inhibition.

**Table 3:** Result of independent T-test for gender and Pearson correlation between age and year of education and study variables.

	M vs F (*P*-value)†	Correlation with age	Correlation with YoE
Age	.68	1	−.34*
Years of education	.89	−.34*	1
Inhibition	.39	.42*	−.20*
Depression	.12	.06	−.22*
Anxiety	.54	.10	−.26*
Stress	.87	−.06	−.15**

YoE = years of education.

† Unpaired *t*-test between the 2 groups.

* *P* < .01.

** *P* < .05.

#### 3.1.2. Voxel-based morphometry

The voxel-based morphometric analysis identified 173 GM volumes involving both cortical and subcortical regions. Pearson correlation analysis indicated that inhibition significantly correlated with 85 brain regions. The detailed correlation analyses are listed in Table 4.

**Table 4:** Correlation table for the relationship between right and left brain volumes and inhibition.

Brain structures	Correlation with inhibition		Brain structures	Correlation with inhibition	
	Right	Left		Right	Left
G_and_S_occipital_inf_volume	−.10	−.01	S_oc_temp_med_and_Lingual_volume	−.12	−.08
G_and_S_paracentral_volume	−.12	−.23**	S_parieto_occipital_volume	−.10	−.14*
G_and_S_subcentral_volume	−.14*	−.08	S_postcentral_volume	−.10	−.09
G_and_S_cingul_Ant_volume	−.21**	−.21**	S_precentral_inf_part_volume	−.04	−.06
G_and_S_cingul_Mid_Ant_volume	−.13*	−.14*	S_precentral_sup_part_volume	−.02	−.07
G_and_S_cingul_Mid_Post_volume	−.13*	−.06	S_temporal_inf_volume	−.12	−.11
G_cuneus_volume	−.04	−.06	S_temporal_sup_volume	−.15*	−.11
G_front_inf_Opercular_volume	−.09	−.12	Cuneus_volume	−.06	−.08
G_front_inf_Orbital_volume	−.09	−.15*	Entorhinal_volume	−.10	−.18**
G_front_inf_Triangul_volume	−.11	−.10	Fusiform_volume	−.16*	−.07
G_front_middle_volume	−.15*	−.20**	Inferiorparietal_volume	−.11	−.14*
G_front_sup_volume	−.16*	−.15*	Inferiortemporal_volume	−.11	−.18**
G_Ins_lg_and_S_cent_ins_volume	−.13*	−.12	Lateraloccipital_volume	−.13*	−.10
G_insular_short_volume	−.03	−.16*	Lateralorbitofrontal_volume	−.14*	−.17**
G_occipital_middle_volume	−.11	−.11	Lingual_volume	−.05	−.11
G_occipital_sup_volume	−.12	−.12	Medialorbitofrontal_volume	−.15*	−.16*
G_oc_temp_lat_fusifor_volume	−.10	−.07	Middletemporal_volume	−.15*	−.11
G_oc_temp_med_Lingual_volume	−.03	−.08	Parahippocampal_volume	−.07	−.14*
G_oc_temp_med_Parahip_volume	−.12	−.16*	Paracentral_volume	−.14*	−.15*
G_orbital_volume	−.13*	−.16*	Parsopercularis_volume	−.09	−.11
G_pariet_inf_Angular_volume	−.09	−.13*	Parsorbitalis_volume	−.11	−.14*
G_pariet_inf_Supramar_volume	−.15*	−.17**	Parstriangularis_volume	−.16*	−.15*
G_parietal_sup_volume	−.09	−.15*	Pericalcarine_volume	−.02	−.08
G_postcentral_volume	−.14*	−.148*	Postcentral_volume	−.12	−.16*
G_precentral_volume	−.12	−.13	Posteriorcingulate_volume	−.11	−.07
G_precuneus_volume	−.16*	−.15*	Precentral_volume	−.11	−.11
G_rectus_volume	−.13*	−.12	Precuneus_volume	−.14*	−.18**
G_temp_sup_G_T_transv_volume	−.11	−.19**	Rostralanteriorcingulate_volume	−.11	−.18**
G_temp_sup_Lateral_volume	−.26**	−.13*	Rostralmiddlefrontal_volume	−.13*	−.22**
G_temp_sup_Plan_polar_volume	−.13*	−.20**	Superiorfrontal_volume	−.19**	−.16*
G_temp_sup_Plan_tempo_volume	−.14*	−.12	Superiorparietal_volume	−.11	−.13*
G_temporal_inf_volume	−.09	−.16*	Superiortemporal_volume	−.21**	−.19**
G_temporal_middle_volume	−.13*	−.10	Supramarginal_volume	−.16*	−.17**
Pole_occipital_volume	−.05	−.05	Insula_volume	−.12	−.13*
Pole_temporal_volume	−.10	−.18**	Cerebellum_Cortex	−.17**	−.21**
S_calcarine_volume	−.10	−.13*	Thalamus	−.22**	−.24**
S_circular_insula_ant_volume	−.16*	−.12	Caudate	−.12	−.12
S_circular_insula_inf_volume	−.06	−.06	Putamen	−.13*	−.15*
S_circular_insula_sup_volume	−.22**	−.12	Pallidum	−.13*	−.14*
S_front_inf_volume	−.07	−.15*	Hippocampus	−.17**	−.23**
S_front_middle_volume	−.09	−.11	Amygdala	−.12	−.25**
S_front_sup_volume	−.14*	−.08	Accumbens_area	−.24**	−.21**
S_occipital_ant_volume	−.05	−.06	Brain_Stem	−.10	−.10
S_oc_temp_lat_volume	−.11	−.05			

Ant = anterior, Cent = central, Front = frontal, G = gyrus, inf = inferior, Lat = lateral, Med = medial, Mid = middle, Pariet = parietal, Post = posterior, S = sulcus, sup = superior, Oc = occipital, Temp = temporal.

* *P* < .05.

** *P* < .01.

To identify the neuroanatomical correlates of depression and anxiety the correlation analysis was performed between GM volumes and anxiety and depression scores. The results revealed a significant negative correlation between depression and GM volumes in 2 brain regions: the left pallidum (*r* = −0.13, *P* = .04) and the right cerebellar cortex (*r* = −0.13, *P* = .04). Additionally, anxiety was found to be negatively correlated with GM volumes in both the left (*r* = −0.13, *P* = .04) and right cerebellar cortex (*r* = −0.15, *P* = .02). Notably, depression and anxiety were not positively correlated with any brain regions. The data suggest that depression and anxiety may be associated with specific brain regions, including the left pallidum and the cortices of both the left and right cerebellum.

#### 3.1.3. Resting-state networks results

Based on the RSN analysis conducted for this study, a total of 9 resting-state networks were identified (see Figure. 2). These networks comprise the anterior default mode network (Ant-DMN), posterior default mode network (post-DMN), SN, right and left frontoparietal networks (R-FPN and L-FPN), FN, VAN, DAN, and thalamus network (ThaN).

To identify the resting-state neural networks associated with depression and anxiety, a correlation analysis was conducted between neural network activation and scores for anxiety and depression. The results showed a positive correlation between depression and anxiety and the thalamus network, with correlation coefficients of *R* = 0.15 (*P* = .02) for depression and *R* = 0.16 (*P* = .02) for anxiety. An additional correlation analysis revealed that the anterior default mode network and the left frontoparietal network exhibited negative correlations with inhibition (*r* = −.18, *P* = .01, *r* = −.13, *P* = .04).

### 3.2. Mediation analysis

To clarify the relationships between variables and elucidate both direct and indirect pathways, a path analysis was performed. In the path analysis, brain structure and resting-state networks were treated as independent variables, whereas depression and anxiety were considered as mediating variables. Inhibition was treated as the dependent variable, with age and years of education serving as control variables. It is noteworthy that before running the path analysis, the correlations between the paths of the initial model were examined and insignificant paths were removed from the final model.

For a more detailed explanation, the initial step involved examining the relationship between depression, anxiety, stress, and inhibition. The results revealed a significant correlation between inhibition and both depression and anxiety (*r* = .15, *P* = .02; *r* = .19, *P* = .00, respectively), whereas no significant correlation was found with stress (*r* = .07, *P* = .28, see Table 2). Therefore, the stress was removed from the next stages of the analysis. The next step involved investigating how depression and anxiety relate to specific brain regions and resting-state networks. The findings revealed that GM volume in the left pallidum and the right cerebellar cortex was associated with depression. Furthermore, there was a correlation between anxiety and the volume of GM in the left and right cerebellar cortices. Consistently, the findings revealed a correlation between depression, anxiety, and the thalamus network. In the final step, path analysis was used to investigate whether depression and anxiety serve as mediators in the relationship between certain brain structures (left pallidum, left and right cerebellum cortex) and the resting-state network (thalamus network), including its impact on inhibition. Table 5 presents the estimates of standardized direct and indirect effects on inhibition.

**Table 5:** Standardized direct and indirect path effects.

Pathway	β	*P*-value	(95% CI)	
			Lower	Upper
*Direct*				
Anxiety → inhibition	.12	.07	−.01	.25
Depression → inhibition	.06	.41	−.09	.23
Left pallidum → depression	−.06	.20	−.16	.03
Left pallidum → inhibition	.05	.44	−.08	.20
Right cerebellum cortex → Anxiety	−.35	.12	−.79	.09
Right cerebellum cortex → depression	.004	.98	−.11	.12
Right cerebellum cortex → inhibition	.33	.10	−.07	.76
Left cerebellum cortex → anxiety	.22	.32	−.21	.67
Left cerebellum cortex →inhibition	−.34	.11	−.76	.08
Thalamus resting-state network → anxiety	.14	**.02**	.02	.26
Thalamus resting-state network → depression	.04	.39	−.05	.13
*Indirect*				
Left pallidum → depression → inhibition	−.001	.26	−.005	.001
Right cerebellum cortex → inhibition (total indirect effect)	−.06	**.05**	−.18	.002
Right cerebellum cortex → anxiety → inhibition	.00	.07	−.001	.00
Right cerebellum cortex → anxiety → depression → inhibition	.00	.24	−.001	.00
Right cerebellum cortex → depression → inhibition	.00	.83	.00	.00
Left cerebellum cortex → inhibition (total indirect effect)	.04	.16	−.02	.14
Left cerebellum cortex → anxiety → inhibition	.00	.16	.00	.001
Left cerebellum cortex → anxiety → depression → inhibition	.00	.27	.00	.001
Thalamus resting-state network → inhibition (total indirect effect)	.03	**.03**	.002	.06
Thalamus resting-state network → anxiety → inhibition	370.38	**.04**	16.46	1112.95
Thalamus resting-state network → anxiety → depression → inhibition	115.81	.30	−134.25	638.01
Thalamus resting-state network → depression → inhibition	55.28	.28	−71.97	484.98

*P < .05.

**P < .01.

***P < .001.

**Figure 3.**
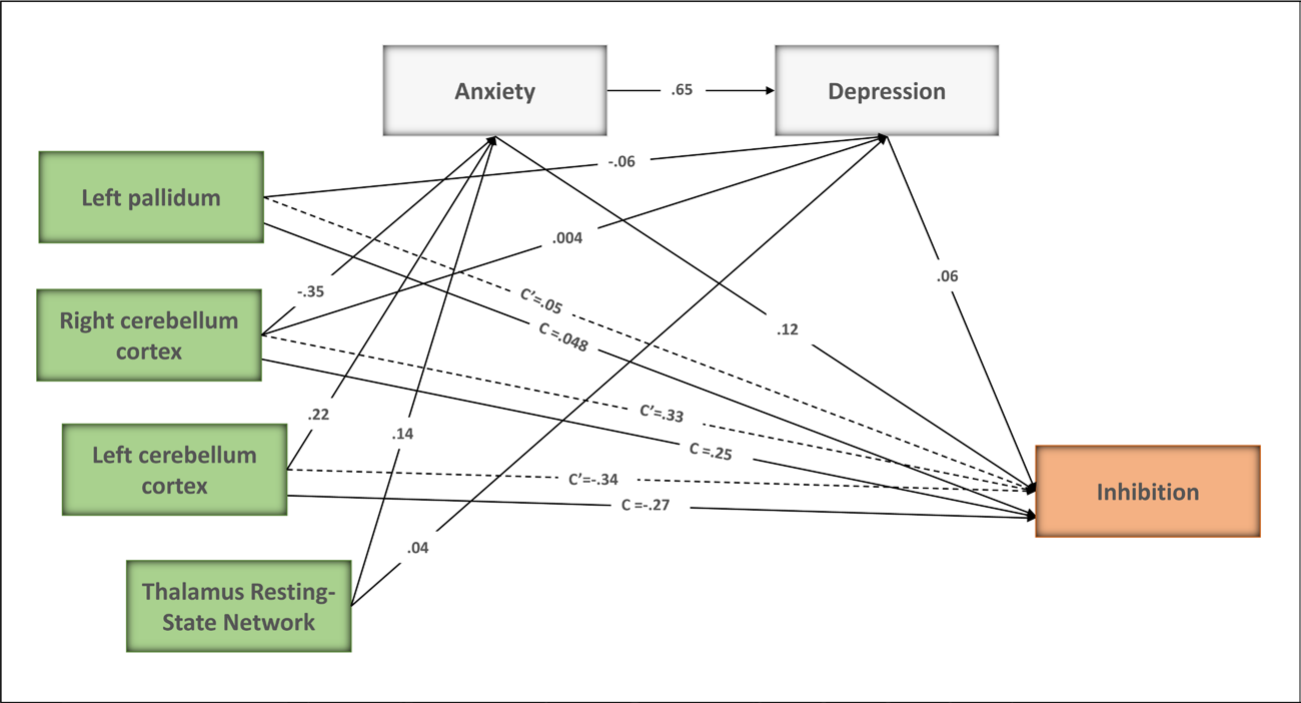
Study model path analysis. The standardized regression coefficients of each path are displayed. The right cerebellum cortex and Thalamus resting-state network through depression and anxiety had a total indirect effect on inhibition. Path c: GM volumes in the left pallidum and the right and left cerebellum cortex are significantly correlated with inhibition. Path c’: after regressing out depression and anxiety, GM volumes in the left pallidum and the right and left cerebellum cortex are significantly correlated with inhibition. GM = gray matter.

The results indicated that the left pallidum (β = .05, *P* = .44), right (β = .33, *P* = .10), and left cerebellum cortex (β = −.34, *P* = .11) had not a direct effect on inhibition. Regarding bootstrap indirect mediation analysis, the right cerebellum cortex (β = −.06, *P* = .05) and thalamic resting-state network (β = .03, *P* = .03) had a total indirect effect on inhibition. Notably, the thalamic resting-state network had an indirect effect on inhibition through anxiety. While the left pallidum (β = −.00, *P* = .26) and left cerebellum cortex (β = .04, *P* = .16) had no significant indirect effect on inhibition. The results of the index analysis suggest that the model fit is satisfactory, as indicated by the following indices: CFI = 0.99, GFI = 0.99, NFI = 0.98, IFI = 0.99, and RMSEA = 0.08, which are detailed in Figure 3.

## 4. Discussion

### 4.1. The summary of the study

This study was performed to examine the effect of depression and anxiety symptoms on cognitive inhibition by using the VBM and RS-fMRI techniques. Behavioral results demonstrated that inhibition was significantly negatively correlated with depression and anxiety. The VBM results showed that depression was negatively correlated with GM volumes in the left pallidum and the right cerebellum cortex. Also, anxiety was negatively correlated with GM volumes in the left and right cerebellum cortex. The RS-fMRI results showed that the thalamus network was positively correlated with depression and anxiety. Furthermore, the anterior default mode network and left frontoparietal network were negatively correlated with inhibition. Finally, mediation analysis revealed that the right cerebellum cortex and thalamic resting-state network through depression and anxiety had a total indirect effect on inhibition.

### 4.2. Cognitive inhibition and depression and anxiety symptoms

Consistent with our results, Burdette et al found that internalizing symptoms (depressive and anxiety symptoms) as indicators of emotional disorders were positively associated with failed inhibition.^[25]^ In a cross-sectional study, McClintock et al reported that the treatment-resistant depressed patients had lower accuracy on the Stroop Test comparing major depressive patients and healthy controls.^[26]^ Further, another study demonstrated that non-melancholic depressed patients were impaired in the Stroop task.^[27]^ A meta-analysis study by Shi et al reported that higher anxiety symptoms were associated with poor attention control.^[7]^ Another study using a computerized Stroop task found a positive association between GAD and impairment in cognitive inhibition. Also, in this study, clinician-rated anxiety severity predicted cognitive inhibition impairment more than the effect of GAD diagnosis, indicating a distinct connection between anxiety and inhibition that is not contingent on a diagnosis of GAD.^[12]^ Contrary to our results, Price & Mohlman showed that the severity of symptoms of GAD symptom severity was associated with better inhibition.^[28]^ Taken together, these findings provide evidence of inhibition deficits in depression and anxiety. Over time, even a small deficit in inhibition may affect a person’s thoughts and lead to persistent and uncontrollable worry and rumination. Focusing on repetitive negative thinking can help perpetuate depression or anxiety and make the situation worse.

### 4.3. Neural correlates of depression, anxiety symptoms

The VBM results showed that depression was negatively correlated with GM volumes in the left pallidum and the right cerebellum cortex. Also, anxiety was negatively correlated with GM volumes in the left and right cerebellum cortex. These findings emphasize the role of the pallidum on the symptoms of depression, as well as the role of the cerebellum on the symptoms of depression and anxiety.

Our findings agree with a recent study that reported a reduction in GM volumes in the left pallidum in the currently depressed patients and remitted MDD patients.^[45]^ The study of Ancelin et al reported a decrease in GM volume in the pallidum in major depressive disorder.^[46]^ Also, volume reduction in the pallidum has been reported in subjects with depressive disorders in a systematic review and meta-analysis.^[47]^ Another study found a disruption in functional connectivity between the left and right pallidum in late adolescents with subthreshold depression.^[48]^ In the study of Kim et al, a positive correlation was shown between the severity of suicidal thoughts in major depressive patients and the level of atrophy in the left pallidum.^[49]^ Since defects in the Cortico-striatal-thalamo-cortical (CSTC) pathway are mainly observed in depression symptoms, considering the importance of the pallidum in this circuit, the role of the pallidum in depression symptoms can be considered.^[50]^ Defects in the CSTC pathway lead to changes in reward signals and thus depressive symptoms.

Consistent with our findings about the role of the cerebellum in depression and anxiety symptoms, recent studies provide evidence about the possible role of the cerebellum in depression and anxiety.^[51,52]^ The research literature on abnormal cerebellar volume in depression and anxiety is heterogeneous. These differences can be the result of measuring different subregions of the cerebellum in the studies. For example, 1 study demonstrated a reduction in GM volume in subregions of the cerebellum in individuals with remitted major depression accompanied by cognitive impairment.^[53]^ On the other hand, in Depping et al study, the volume of GM in the affective/limbic cerebellum increased in patients with major depression during electroconvulsive therapy.^[54]^ Conversely, in the study by Bogoian et al, a positive relationship was observed between the severity of depressive symptoms and the volume of the vermis in the cerebellum.^[55]^ Similarly, in the study by Talati et al, Increased GM in the left cerebellum was observed in patients with social anxiety disorder.^[56]^ In general, studies on the role of the cerebellum in emotional disorders have been conducted in various fields such as electrical stimulation studies, genetic models, or brain lesions. Taken together, the evidence emphasizes not only the role of the cerebellum in motor processes but also in cognitive and emotional processes.^[57]^

Furthermore, the RS-fMRI results showed that the thalamus network was positively correlated with depression and anxiety. The thalamus has been considered as a vital relay center of the brain in the flow of sensory information to the cerebral cortex.^[58]^ Several studies have emphasized the importance of the thalamus in anxiety and depression. For example, a recent review study shows that in major depressive disorder, the most important causal center that needs intervention is the thalamus.^[59]^ Also, another study reported a significant volume reduction in the left thalamus in the early stages of major depressive disorder.^[60]^ Similarly, in the Qiao et al study, patients with GAD showed stronger functional connectivity in the thalamus.^[61]^ Also, a study by Y. Zhang et al using macro-micro neuroimaging techniques clarified the central role of the thalamus in the structural abnormalities of depression.^[62]^ Contrary to our finding, which only shows the involvement of the thalamic resting-state network in depression and anxiety symptoms, previous research has shown the involvement of main brain networks, including the default state network, the central executive network, and the SN.^[23,63,64]^ Our findings only highlight the involvement of the thalamic network as an important region of the limbic system in depression and anxiety symptoms. Considering the relationship of the thalamus with the structures of the limbic system and its involvement in cognitive and emotional processes,^[65]^ its involvement in the symptoms of anxiety and depression is not out of mind.

### 4.4. The mediation effect of depression and anxiety

Mediation analysis revealed that the right cerebellum cortex and thalamic resting-state network through depression and anxiety had a total indirect effect on inhibition. It is estimated that more than half of the cerebellar cortex as a vital component in the human brain is connected with the cortical communication areas.^[66]^ In addition to the precise regulation of motor activity, the cerebellum plays a role in regulating many different functional characteristics, from executive function such as inhibition^[67]^ to mood regulation.^[52]^ Our findings show that the cerebellum affects cognitive inhibition by mediating depression and anxiety. The importance of these findings is evident when considering the reduction of GM volumes in the cerebellum cortex in depression and anxiety which are characterized behaviorally by emotion processing impairments.

On the other hand, the thalamus also has numerous functional connections with other brain regions. The importance of the thalamus as a conduit for information transmission in the brain is due to its multiple roles, including information filtering, role in the process of cognitive function, and regulation of emotions.^[68]^ Our findings show that the thalamic network influences cognitive inhibition by mediating depression and anxiety. Considering the role of the thalamus network in filtering information, it can be said that there may be too much sensory information entering the brain in the symptoms of depression and anxiety. In other words, information suppression is not enough to limit negative content in depression and anxiety symptoms.

## 5. Limitations

The current research had limitations and possible factors that need further investigation. First, in the present study, The DASS was adopted to evaluate the symptoms of depression and anxiety. It is suggested that clinical interview be used in future studies to obtain a more reliable and accurate of depression and anxiety symptoms. Second, in this research, the causal relationship between depression and anxiety symptoms and brain changes has not been investigated. For future directions, longitudinal studies can be developed to determine the causal relationship. Thirdly, in this research, VBM and resting-state functional MRI techniques were used to investigate neural correlates of depression and anxiety symptoms. For future studies, it will be important to use multimodal brain imaging measures to obtain a more accurate measure of neural correlates related to depression and anxiety symptoms.

## 6. Conclusion

In conclusion, the present study using the structural MRI and resting fMRI techniques implied neural substrates of the effect of depression and anxiety symptoms on cognitive inhibition. Unique to this study was the finding that the right cerebellum cortex and thalamic resting-state network through depression and anxiety had a total indirect effect on inhibition. To sum up, elucidating the neural mechanisms behind the detrimental effects of depressive and anxious symptoms on cognitive inhibition may be essential for designing strategies aimed at enhancing individuals’ cognitive well-being.

## Acknowledgments

This article relates to the dissertation of Maede Bahri for a Ph.D. degree in Neuroscience (Grant number: 57991) at the School of Advanced Technologies in Medicine of Tehran University of Medical Sciences. We are grateful for the support and contributions of Perplexity AI in proofreading and refining the manuscript.

## Author contributions

**Conceptualization:** Maede Bahri, Hassan Farrahi, Seyed Amir Hossein Batouli.

**Data curation:** Maede Bahri, Maryam Bahri, Hami Mahdavinataj, Seyed Amir Hossein Batouli.

**Formal analysis:** Maede Bahri, Maryam Bahri, Hami Mahdavinataj, Seyed Amir Hossein Batouli.

**Funding acquisition:** Seyed Amir Hossein Batouli.

**Methodology:** Maede Bahri, Hassan Farrahi, Maryam Bahri.

**Project administration:** Seyed Amir Hossein Batouli.

**Supervision:** Hassan Farrahi, Seyed Amir Hossein Batouli.

**Validation:** Seyed Amir Hossein Batouli.

**Writing – original draft:** Maede Bahri.

**Writing – review & editing:** Maede Bahri, Hassan Farrahi, Maryam Bahri, Hami Mahdavinataj, Seyed Amir Hossein Batouli.
